# Using normalisation process theory to evaluate the implementation of a digital health intervention in community and secondary care long COVID clinics

**DOI:** 10.1136/bmjopen-2024-092824

**Published:** 2024-11-27

**Authors:** Fiona A Stevenson, Paul Pfeffer, Sarah Walker, Hadiza Ismaila, Vinosh Jegatheesan, Ibrahim Mohammad, Ann Blandford, Stuart Linke, John R Hurst, William Ricketts, Fiona L Hamilton, David Sunkersing, Katherine Bradbury, Henry Goodfellow

**Affiliations:** 1 Research Department of Primary Care and Population Health, University College London, London, UK; 2 Department of Respiratory Medicine, Barts Health NHS Trust, London, UK; 3 University of Exeter Medical School, Exeter, UK; 4 University College London, London, UK; 5 UCLIC, University College London, London, UK; 6 Academic Unit of Respiratory Medicine, UCL Medical School, London, UK; 7 Respiratory Medicine, Barts Health NHS Trust, London, UK; 8 Institute of Health Informatics, University College London, London, UK; 9 Psychology, University of Southampton, Southampton, UK

**Keywords:** COVID-19, Digital Technology, eHealth, Organisation of health services, QUALITATIVE RESEARCH

## Abstract

**Abstract:**

**Objectives:**

The potential and expected benefits of digital health interventions (DHI) have long been discussed, yet substantial challenges are associated with deploying DHI at scale. Insights are presented concerning the implementation of a DHI consisting of a patient-facing app and a digital dashboard for clinicians providing supported self-management for long COVID to support both clinicians and patients.

**Design:**

Qualitative reflexive thematic analysis, mapped against Normalisation Process Theory.

**Setting:**

Fifty-five and a half hours of zoom recordings of meetings between clinicians in community and secondary care long COVID clinics and members of the research team.

**Participants:**

Allied health professionals, service delivery managers and members of the core team, including representatives from industry partners.

**Results:**

The DHI fitted with contextual circumstances and the design supported flexibility to suit circumstances in different trusts. The DHI also aligned with existing ways of working.

Healthcare professionals worked together to support the implementation of the DHI, requiring flexibility to take account of local circumstances. The DHI was appraised in both positive and negative terms by healthcare professionals. Using DHIs was said to have the potential to complement care but not be a replacement for face-to-face clinical input. The DHI was judged to have demonstrated the potential to affect long-established patterns and organisational structures of engagement between healthcare professionals and patients in terms of access to care.

**Conclusions:**

NPT provided a framework for considering both individual agency and the organisation context, enabling reflections to be made at the level of the structure of services as well as people’s experiences. The discipline of considering first the context, then the work and finally the practical effects helped place order on the ‘mess’ involved in the rapid cycle of developing, refining and implementing a DHI in an atypical environment (a pandemic).

**Strengths and limitations of this study**

- We present an analysis of discussions captured as a clinically supported self-management digital health intervention (DHI) was developed and implemented.
- We could not observe conversations between clinicians and patients to understand how the DHI was introduced and use negotiated on a day-to-day basis.
- We also only have accounts of what people said they did with no observation data on, for example, how clinicians managed the clinical dashboard to support patient care despite this being a key aspect of the DHI.
- The voices represented are overwhelmingly those who were committed to a digital solution.

## Background

 The potential and expected benefits of digital health interventions (DHI) have long been discussed. However, the UK NHS has recently been judged to be ‘in the foothills of digital transformation’ with the benefits not fully realised and interventions adding to the workload of clinicians rather than releasing more time to care.^1^ Moreover, there are substantial challenges with deploying DHI at scale^,^.^2^,^4^ The COVID-19 pandemic resulted in the rapid adoption of digital technology in the NHS and significant changes in the development, reconfiguration and delivery of services more widely.^5^ These rapid changes have shown what is possible, although recent data indicate that, postpandemic, most appointments are again being delivered face-to-face.^6^

The Living With COVID Recovery (LWCR) DHI was set up during the pandemic to provide evidence-based interventions for the long-lasting symptoms of COVID-19 infection,^7^ often referred to as long COVID.^8 9^ It consists of three main components: (1) a clinical pathway through which patients access the programme after clinical assessment for safety and suitability, (2) a patient-facing app delivering structured, integrated treatments and advice under allied health professional (AHP) supervision and collecting patient-reported outcome measures (PROMs) and (3) a digital dashboard for clinicians (which displays PROMs and allows AHP rapid review of patients). The DHI targets the main symptoms patients reported experiencing for weeks and even months after the peak of their acute infection (eg, fatigue, anxiety and breathlessness). It combines evidence-based resources from physiotherapists, occupational therapists, GPs, psychologists, dietitians and respiratory physicians to create bespoke treatment plans for each patient.^7^

The DHI was developed by researchers, software developers and clinicians working directly with COVID/long COVID patients.^10^ Meetings identified the challenges being faced by clinicians and patients with COVID during the pandemic, and the clinical needs that required addressing defining the areas of focus for the DHI.

Following a clinical assessment for safety and suitability, patients were signed up to use the DHI (‘onboarded’) by clinical staff, generally an AHP. The DHI took the form of a smart phone application (App) downloaded on their phones. Patients could access the App as and when they need it but could also be directed by clinical staff to review specific sections to support individual symptoms or problems. Given the novelty of the clinical area (long COVID) and differences in how NHS clinics were organised to support patients, the DHI was designed to be flexible to fit with structures and needs of the clinics. Flexibility and iterative use and design were key to the development and implementation of the DHI to maximise utility and use. Iterative development and tailoring of the DHI incurred no costs for local services.

The LWCR DHI exemplifies two potential benefits of digital health: (1) enabling healthcare practitioners (HCPs) to manage larger numbers of patients than would otherwise be possible and (2) a patient-centred, accessible and convenient service to patients. This paper reflects on the experiences, including challenges, of multidisciplinary working across complex organisations (NHS/Universities) alongside industry partners (software developers) to solve a real-time medical problem (long COVID) about which awareness and the associated knowledge base were continuously evolving. The project was funded from October 2020 to September 2022. At the time of the final collection of data drawn on in this paper (April 2022), the DHI was active in 31 clinics with 3754 patients registered.^11^

We present insights concerning the implementation of LWCR DHI into clinical care to support both clinicians and patients, while simultaneously offering opportunities for more efficient delivery of services. Specifically, we were interested in how decisions in relation to implementation of a DHI providing supported self-management for long COVID into clinical care to support both clinicians and patients were negotiated and sustained.

## Methods

### Data

Data comprised 55.5 hours of recorded Zoom^12^ meetings: 43.5 hours of meetings between clinicians and members of the research team to understand experiences of implementing the DHI in practice, 2 hour-long meetings about study set up with two major trusts and 10 hours of meetings with the core team relating to problem solving and technical issues (table 1). All meetings lasted 1 hour, with the exception of one between clinicians and members of the research team that lasted 90 min.

**Table 1:** Data used for analysis

Meeting purpose	Attendees	Number of meetings	Date ranges
Onboarding and development	Major trusts	2	06/2020−10/20202
Problem solving and technical issues	Project team	10	05/2020−05/2021
Understanding experiences of implementing the DHI in practice	Discussions between clinicians, and members of project team	43.5	04/2020−04/2022

DHIdigital health interventions

The 43.5 hours of meetings about implementation took place over 2 years (April 2020−April 2022) at all stages of the development of the DHI from the initial discussions through to how the DHI worked in practice. Recordings included a key informant interview with the physiotherapist who first implemented and supported the rollout of the DHI. Clinicians were mostly AHPs (physiotherapists, occupational therapists, speech and language therapists, psychologists), service delivery managers and members of the core team (clinical academics, research academics, and representatives from industry partners).

### Patient and public involvement

Patient and public involvement (PPI) was key to the study which involved identifying user requirements in the context of a poorly understood condition and emerging clinical pathways. We asked PPI to advise around content, functionality, onboarding and clinical pathways in relation to the DHI. We had 30 PPI representatives involved over the course of the whole project. All meetings took place on Zoom, with payment provided for prework and attendance at meetings. From the outset of the project, there were two PPI representatives on the study steering group actively participating in monthly meetings. Each of the work package management groups included two PPI representatives, one of whom contributed to discussions that helped shape this paper and whose contribution is acknowledged. PPI representatives were not involved in the analysis presented here as the project had finished.

### Analysis

Initial discussions were held within the implementation work package meetings to consider how best to understand implementation of the DHI. A core group of five (FAS, DS, EM, VJ and IM) examined three recordings and transcripts and met on multiple occasions to discuss initial ideas and resolve any differences of interpretation. All recordings were then reviewed by the first author, who conducted coding across the whole data set, taking into account earlier discussions regarding the data. Initial analysis was conducted using the principles of reflexive thematic analysis,^13^ with themes subsequently mapped against the constructs of Normalisation Process Theory (NPT).^14^ The wider project team reviewed the analysis, and changes were made by the first author.

NPT as an established theory of implementation developed as a tool to explain and evaluate the processes that shape the translation of innovations in the organisation and delivery of healthcare.^14^ It offers a coherent and stable set of explanations of implementation processes and characterises the mechanisms that motivate and shape these processes and so can be used to evaluate and understand implementation processes.^15^ Its use was planned from the inception of the study and shaped data collection and analytic direction throughout the project by providing a structure to support capturing of changes in the way people thought about and used the DHI as it was implemented into practice that could then be used to further iterate the DHI for future users.

NPT consists of 12 primary constructs, organised according to the Context–Mechanism–Outcome configuration employed in realist evaluation studies. It provides a structure for analysis according to (1) implementation contexts: the settings in which implementation work is done and how possibilities are shaped by the environment, (2) implementation mechanisms: the work that people do when they participate in implementation processes and (3) implementation outcomes: how things change when interventions are implemented.^14^ Within each of these domains, there are 12 NPT constructs (four for each of the Context–Mechanism–Outcome domains). Details are included in online supplemental appendix 1.

Using NPT provided a framework to build on existing knowledge and theory relating to implementation, as well as an opportunity to consider the robustness of the tenets of NPT in relation to the extraordinary circumstances and organisational pressures and changes during the COVID pandemic.

## Results

### Implementation contexts

The DHI was introduced to address a condition (subsequently labelled long COVID) emerging as a set of symptoms in a service under extreme stress, staffed by overstretched clinicians.^16^,^18^ It was striking that some of the clinical contexts appeared relatively stable, while others were in a constant state of flux, with staff members seconded for variable numbers of days a week from other settings into the long COVID clinics.

The DHI aimed to use evidence-based, digitally delivered rehabilitation material to support people remotely. A key goal was to validate patients’ experiences in a context when it was often denied or obfuscated and to make patients feel cared for. Crucially, the DHI aspired to reduce the strain on the UK National Health Service (NHS).

It is important to acknowledge that another product was being developed in parallel with LWCR by a different team but also funded through NIHR: a web-based product called Your COVID Recovery (YCR). Both YCR and LWCR were free for the duration of the study funding, but YCR was publicised to NHS trusts through briefings via NHS England.

NPT highlights the importance of context in shaping the formulation and planning of interventions and their components (the construct of *strategic intentions*). The LWCR DHI was delivered via a smart phone application. This fitted with minimising contact between people in a pandemic but also with the Department of Health policy agenda of ‘Digital First’; that digital technology should be used to enhance the precision, personalisation and efficient delivery of care.^19 20^ The focus on self-management of care aligned with the ongoing focus on increasing self-management that predated the pandemic.^19^ The focus on self-management was seen as a particularly attractive feature by clinical staff.

[the] big unmet need is supporting patient self-management … patients to be responsible for their own, to support their own recovery-totally behind that, real, real need for that. (Doctor, November 2020 meeting with clinician introducing LWCR)

It is important to note that at the time of development and implementation of the LWCR DHI, there was a lot of discussion within the wider long COVID community on social media about what should be offered and specifically whether the focus should be on self-management. Part of the background to this was historic hostility against reconditioning from the community with chronic fatigue syndrome (CFS). Although the team were always clear that long COVID was not the same as CFS, this debate influenced both the development team and clinicians in clinics.

Differing contexts affect the ways in which users can find and enact workarounds that make an intervention and its components a workable proposition in practice (a*daptive execution*). Crucially, LWCR not only targeted an unmet clinical need, but one that was seen as important to meet. It did not replace/adapt something that existed but rather supported the creation of new clinics for which a case for funding could be made.

LWCR had to be established in each trust individually; however, there was large variability in the processes required across trusts. In some trusts gaining information governance approvals, procurement and contracts, along with signing Terms and Conditions was described as ‘the fundamental roadblock*’*. However, the combination of the need and the context of operating in an ‘emergency’ in some cases led to a more streamlined journey through information governance and other systems. In some trusts, delays in the processes necessary to use the DHI led to workarounds such as sending out paper versions of the questionnaires on the DHI so staff could start supporting patients while waiting for formal sign off.

The NPT construct *negotiating capacity* considers how contexts affect the extent to which an intervention and its components can fit or be integrated into existing ways of working by their users. The DHI was presented as a core part of NHS clinical care integrated into the care pathway, in line with national guidelines. It was developed by an experienced multidisciplinary team with academic, clinical and industry expertise. Several of the team had previously worked with the developer (Living With) making the company and their solution a known quantity.

Finally, we consider how existing social structural and social cognitive resources shape the implementation environment (*reframing institutional logics*). The intensity of work necessary to develop and deliver an intervention at speed to deal with a global emergency meant compression of processes relating to implementation, such as building partnerships across teams who had not previously worked together. For example, in one trust, physiotherapists were invited to make the case, based on their experience, for using the DHI. Notably, the meeting started with the chair stating the purpose of the meeting was to decide whether to adopt LWCR, presenting this as a decision to use LWCR as opposed to YCR.

In [trust] we have a decision to make to take this sort of narrow and stony path of going with a different app compared to the national picture rather than the broad path of the generic COVID recovery [YCR] that the rest of the country is doing. (Clinical lead, Trust meeting October 2020)

This is an example of the cross-disciplinary work that was key to establishing the legitimacy of the DHI. The chair presented the difficulties of using LWCR, ‘a narrow and stony path’ compared with YCR ‘the broad path of the generic COVID recovery’; however recommendations from the physiotherapists led to the LWCR DHI being adopted.

### Implementation mechanisms

Key to enacting interventions is how people work together to understand and plan the activities that need to be accomplished to put an intervention into practice (*coherence building*). Understanding the value of an intervention is key to implementation. The DHI did not duplicate existing services and was clearly presented as providing digital support as opposed to a challenge to the current services and how they were delivered then and in the future. There was however a difference in how clinics used LWCR. Some clinics used it to help them structure their clinic from the outset and built their service provision around it, whereas others treated it as an adjunct and did not fully exploit all the features.

The LWCR DHI was designed to support an identified space where work was not being directed, namely recovery. This contrasted with the major focus of clinical work at the time which was dealing with acutely ill patients. It was presented as reducing workload, with a key ‘selling point’ being that initial implementation by a physiotherapist indicated that it took on average between two and two and a half min per patient to oversee and manage care. This meant optimal use could be made of scarce staff resource.

The focus of the DHI on providing supported self-management and rehabilitation, the notion of ‘trickling support’, was presented as tackling feelings of abandonment. Crucially, it was stressed by the software developers that implementing the DHI was not about giving people the DHI and telling them to get on with it, but rather people feeling supported by clinical staff to manage their symptoms. It was stressed that this was particularly important with long COVID where the evidence base was still developing.

… the power of a tiny bit of clinician connectivity is worth its weight in gold. You know, it’s may be always true of any condition, but I don't think so, because nobody knows anything about long COVID. It’s really, really powerful. (Software developer, presentation to a trust, November 2020)

A key aspect stressed throughout when introducing the DHI was the flexibility around the implementation to fit with existing pathways and provision of services. Each service was invited to tailor use of the DHI and develop a clinical pathway that best-fitted their needs. Clinicians were also invited to contribute to a feedback loop for iterative development in the context of a rapidly evolving understanding of long COVID, allowing them to shape the DHI and use it in ways that best suited their clinical environment and the organisation of their services. This feedback was used by the team, and also shared by the core team with other possible users interested in implementing the DHI into their services by providing ‘on the ground’ experiences.

The interaction between the development of a product to provide a service alongside the development of robust research evidence to understand the mechanisms of action and provide data to support future development and use of the DHI caused tension in relation to *coherence building*. This related to the quantity and nature of PROMs collected from patients as part of the DHI, with a balance needing to be reached between those necessary for clinical management, those needed for evaluation and research and ensuring completion by a population whose symptoms generally included ‘brain fog’ and cognitive fatigue.

Overall, work involved in implementing the LWCR DHI in practice was summed up by one of the software developers as needing to ‘embrace the mess’.

It is also important to consider how people worked to create networks to build and sustain a community of practice around the DHI (*cognitive participation*). Key to this was the idea of boundaries of clinical responsibility for patient care, not just between clinicians but also between clinicians and patients. The DHI was designed to provide ‘supported self-management’, providing patients with the autonomy to manage their own care within the bounds of clinical oversight. Management of this in practice formed a point of intense discussion between AHPs who had adopted the DHI and their colleagues relating to giving patients access to information in the DHI which was not filtered on a patient-by-patient basis by a medical professional. This suggests that one barrier to patient self-management may lie with healthcare professional paternalism, likely driven by uncertainty about how best to support patients under their care.

A central pillar of the LWCR DHI is patient self-management. The offer of a DHI was presented as potentially delicate with concern that people may feel they were being ‘fobbed off’ and not provided with care that was overseen by healthcare professionals.

if we say to people, ‘We’re going to call you in two weeks’ time and before then, we really want you to fill out these questionnaires because then we’ll talk through your results and we’ll talk about your goals and we’ll link it all in’. Then they’re like, ‘OK, well, there is purpose here. I’m not just being shoved onto an app’. Someone actually said to me, ‘Is there a human being monitoring the dashboard or is it a robot?’ I was like, ‘No, it’s actually people’. And they were like, ‘Well, no, that’s amazing, if it’s people then we’re happy to do it’. But I think they don’t want to think that their data’s just going into the abyss, do they? (Specialist physiotherapist, trust meeting, May 2021)

The DHI allowed the team to track whether people were getting better or deteriorating based on data recorded by patients. This was seen to help guide conversations with patients to make consultations more productive based on work done by patients between consultations.

We have very rarely been able to really truly say, you’re in charge of your own condition and I think the app allows us to do that. (Specialist physiotherapist, catch up with AHP in trust meeting, November 2021)

This led to a description of the role of the AHP as supporting as opposed to leading care:

Standing alongside and cheer leading (AHP, clinician feedback meeting, Nov 2020))

The examples above demonstrate the beginnings of a change in the delivery of care in terms of the relationship between patients and healthcare professionals. Yet, this was not uniformly reported, with the suggestion of a need for a change in mindset by some clinicians to recognise the value of providing care using supported self-management via DHIs.

Us throwing people onto an app because we're too busy to see them, which is the perception I think of some clinicians which I do understand. (…) convincing these clinicians that actually this isn't a second-rate option, this is a brilliant way for us to manage a big group of people really well and that’s where I think that there’s this sort of it’s a real change in mindset for us. (AHP, trust feedback meeting February 2021)

Similarly, some clinicians reflected that self-management was not seen as palatable by patients, who wanted a cure as opposed to management.

Having considered formation of communities of practice around the implementation of the DHI, we now reflect on the work people reported that they did together to implement the DHI in practice (*collective action*).

The lack of joined up funding between community and secondary care and a prohibition on removing resources from current rehabilitation services was used as leverage by some trusts to recruit additional temporary staff, often AHPs, to support the DHI. However, limited administrative support meant that the administrative work of offering and signing people up to the DHI, once they had been medically assessed as suitable, was undertaken by AHPs. AHPs argued that in practice this provided added value in terms of what could be offered clinically in presenting this as a medically legitimate and supported service. This in turn was judged to increase the therapeutic value and increase patient buy-in.

sometimes we’re the first person, clinical person who’s heard it. So being able to listen and validate, let people know that they’re not alone, that’s such an important thing for us to be doing and we need to spend as much time doing that as we can. (AHP, feedback at meeting with trust November 2021)

Further evidence for the increased value of clinical input in signing patients up to the DHI was demonstrated early in the introduction of the DHI when sending out 800 letters at one trust offering the DHI to people affected by long COVID resulted in only eight sign ups to the DHI.

Finally, it is important to consider how people worked together to appraise the DHI (*Reflexive monitoring*). Reflections in interviews with providers who had implemented the DHI suggested that for populations who are ‘internet savvy’, it worked well. Although it was also suggested that the DHI ‘was kind of a scratching the surface’, particularly in cases in which people had already tried to self-manage using the internet.

Interviews with providers in areas with mixed levels of deprivation reflected that although the necessary physical equipment, in terms of digital devices, can be provided, this does not address the needs of those who do not want to engage with digital care because they do not trust it and/or do not want to engage with digital technology. Concerns were expressed around being able to support those without access to the DHI to ensure that they received an equitable service. This chimes with ongoing concerns around digital exclusion.^21^

Finally, it was suggested that if the DHI was to continue there is a need to demonstrate cost effectiveness.

[We] need to be showing that we’re making effective use of taxpayers’ money and really working through our waiting lists and demonstrating improvements in waiting times. (AHP, feedback at meeting with trust November 2021)

### Implementation outcomes

Finally, we consider what has changed following implementation of the LWCR DHI. First, what practices have changed (*intervention performance*).

The initial focus when developing the DHI was on patient discharge and how and when this would be achieved. This focus shifted over time to the DHI operating as a means of communication and acting as a safety net for patients who were too well to be seen in clinics but not well enough for clinicians to feel comfortable discharging them completely. The DHI therefore functioned to support people in a way that had not been foreseen or intended.

Key throughout the development and evaluation of the LWCR DHI was the inability to integrate data from the DHI into the medical record. This was a cause of frustration for both the project team and the clinical staff. This is an ongoing issue in the implementation of DHIs in the UK NHS due to the use of different information technology systems, concerns about data security and access and concerns about who should be able to amend medical records.

LWCR DHI was presented as having the potential to affect long-established structures of engagement between healthcare professionals and patients enabling patients to access the health service as they need it as opposed to according to appointment times set by the healthcare system (*relational restructuring*).

I think in the past it’s been very much, sort of, patients go to clinicians to tell them what to do and in this sense we’re saying, here are all the tools, we’re here to coach you through it, (…) And they can get in touch with us whenever they like if they’ve got questions, and that I think is the unique, kind of, what this brings to a service (…)

So for me I think it’s about ownership and changing the focus of control and allowing people to engage and to change at a pace that suits them as opposed to what suits the healthcare system (Specialist physiotherapist, follow up interview with provider November 2021)

The introduction of the DHI could also be seen to have changed the ways services are traditionally delivered (*normative restructuring*). The focus here was around changes to the traditional medical pathways in which reconsultation appointments are generally set according to the needs of the service to a system enabled by the DHI of greater responsiveness to patient need for care.

(…) actually traditional medical pathways are very un-patient friendly. The idea that we’ll see you at three months and it doesn’t matter what happens in the next three months. You might become cured or you might be in ITU (intensive care unit), it will be three months till your next appointment, is a ridiculous system. And actually building these patient-responsive pathways, where we move people forward if they’re not doing well, and actually move people back if they’re doing well and say, ‘Actually, you don’t need to be seen again, you’re doing well’, I think is a big directional change in the NHS. (AHP collaborative workshop April 2021)

In relation to *sustainment (normalisation*), HCPs reflected on the increased adoption of digital resources over a short period, suggesting increased agility and flexibility in systems due to COVID had enabled innovation, with hopes this could become the ‘new normal’.

However, limitations of digital interventions were also made clear, with comments that a DHI can complement care, but not be the core, and is not a replacement for direct clinical input. This is in keeping with the idea of the need to carefully consider the place of digital resources in the healthcare system.^22^

But I do try and, in a way, push but being very clear that this isn’t a replacement for input from clinicians, this is a resource to use. (AHP feedback November 2021)

## Discussion

This paper analyses the implementation of the LWCR DHI, a supported self-management intervention for long COVID by a multidisciplinary team during the COVID pandemic. The analysis uses NPT as a lens through which to organise and consider data, consisting of recordings of remotely conducted meetings, from initial development to feedback on the implementation by clinicians using the DHI.

We found that LWCR DHI was timely and fitted to the contextual circumstances: a pandemic in which physical contact needed to be limited as part of a healthcare service which was struggling to offer support for people who were not acutely ill (*strategic intentions*). There was, however, another product aimed at providing support for people with long COVID (YCR) and therefore adoption of LWCR required a ‘leap of faith’.

The design of the DHI supported flexibility to suit circumstances in different trusts, enabling workarounds as necessary, and opportunity to shape the product through feedback (*adaptive execution*). The range of expertise supporting the development of the DHI enabled it to be fitted into existing ways of working (*negotiating capacity*). Given the need, practitioners from different medical disciplines worked across boundaries to make decisions to facilitate adoption and implementation (*reframing organisational logics*).

People worked together to move the LWCR DHI into practice based on agreement as to the place of the DHI supporting delivery of care for an identified gap in service (*coherence building*), as well as an opportunity to shape care in the future. Discussions about implementation demonstrated the need to establish ways of working that fitted with the underlying principles of the LWCR DHI of supported self-management. Also, the discussions revealed how the delivery of care in this way could be both welcomed and experienced as challenging by both practitioners and patients (*cognitive participation*). Personal contact and awareness that the DHI was supported by healthcare professionals was important for both uptake and engagement.

Work to implement the DHI was not always straightforward and required flexibility to take account of local circumstances (*collective action*). The DHI was appraised in both positive and negative terms in relation to access to digital resources (*reflexive monitoring*). Interestingly, the DHI was negatively appraised in relation to people who had a high level of digital literacy as it was felt that it provided limited added value to these patients, despite the facility to contact healthcare professionals.

An unintended function was that the DHI provided support for people in the space between care and discharge.

The LWCR DHI was presented as having the potential to affect long-established structures of engagement between healthcare professionals and patients in terms of access to care (*relational restructuring),* as well as how services could be delivered (*normative restructuring*). It was not, however, possible to directly integrate information collected from the DHI into the patient record, a pre-existing issue with DHIs that remains to be solved (*intervention performance*). Finally, reflections were made in both positive and negative terms about the future of DHIs (*sustainment (normalisation*)).

Use of theory provided the tools for thinking about introducing a digital solution to manage a condition, long COVID, in a fast-evolving situation in which everyone’s understanding was changing rapidly. We used NPT as it has been shown to offer a coherent and stable set of explanations of implementation processes.^15^ It provided a framework for considering both individual agency as well the organisation context, enabling reflections to be made at the level of the structure of services as well as people’s experiences. The discipline provided by using NPT of considering first the context, then the work and finally the practical effects helped place order on the ‘mess’ involved in the rapid cycle of developing, refining, and implementing a DHI in an atypical environment (a pandemic).

It is important to note that clinics and trusts were encouraged to implement the DHI in the way that made sense with their existing services, so although the overall context of a pandemic was a constant, there was no single ‘implementation’. For example, some clinics tried to use as many features of the DHI as possible and encouraged patients to complete PROMs on an ongoing basis, while other clinics just sought data at baseline as the minimum required by trusts. Although the analysis demonstrates areas in which things could change, such as how services could be delivered differently, certain aspects remain intractable such as the difficulty of managing information governance, contracts and data sharing agreements in a timely way and being able to integrate information collected from patients as part of a DHI into the patient record.

### Strengths and limitations of the methodology

The analysis was based on recordings of meetings to discuss the development and implementation of the DHI. This allowed us to capture interactions between people and develop an understanding of how development and use of the DHI was negotiated independently of people’s accounts of what happened. However, restrictions related to the COVID-19 pandemic meant that we were unable to observe conversations between clinicians and patients to understand how the DHI was introduced and use negotiated on a day-to-day basis. We have no observation data on, for example, how clinicians managed the clinical dashboard to support patient care despite this being a key aspect of the DHI. Finally, it is important to note that overwhelmingly our data represent those who advocated for the DHI, and we have few voices from those not committed to a digital solution.

## Conclusions

Using the tenets of NPT helped us understand and elucidate the organisational changes necessary to implement a DHI to support people with long COVID while understanding of long COVID was evolving, and the health service was experiencing extreme organisational stress.

We demonstrated how success in implementing a DHI depends on understanding the complexity of the context, fostering cohesive teamwork for execution of the implementation and evaluating tangible changes resulting from the intervention.

NPT helped us frame our analysis to understand the requirement for people to work differently at all levels of the organisation to implement the LWCR DHI. We were able to capture the ‘workarounds’ people used and the necessity of champions, but also a willingness to engage people across different disciplines to work together to make implementation happen.

Crucially, even in times of extreme organisational change, issues such as managing information governance and problems with integrating data collected via the DHI into patient records remained. Analysis demonstrated the fragmentation of clinical services and differing possibilities across NHS services, with negotiation necessary at the level of each trust reflecting the difficulties, even in times of crisis, of implementing services at speed and scale. This paper therefore provides an opportunity to reflect on entrenched issues with implementation associated with the fragmentation of the UK health service.

### Contributions to the literature

- The COVID pandemic provided a serendipitous opportunity to develop and implement digital health interventions.
- The need to work differently in the pandemic meant people were prepared to try different solutions to the delivery of healthcare.
- Systems in relation to information governance and data sharing eased in some trusts, but not all, and ongoing issues such as integration of data from digital interventions with patient records remained intractable.
- The underlying fragmentation of the UK NHS remains a barrier to implementation.

## supplementary material

10.1136/bmjopen-2024-092824 online supplemental file 1

## Data Availability

Data are available upon reasonable request.
