# Supplementary material for: Using normalisation process theory to evaluate the implementation of a digital health intervention in community and secondary care long COVID clinics
Source: BMJ Open. 2024 Nov 27;14(11):e092824. doi: 10.1136/bmjopen-2024-092824 (PMC11603829; doi:10.1136/bmjopen-2024-092824)
Supplement: online supplemental file 1 [file bmjopen-14-11-s001.docx]

**Appendix 1 - Domains of Normalisation Process Theory (adapted from May et al 2022)**

Implementation contexts

1. Strategic intentions – how contexts shape the **formulation** and **planning** of interventions and their components.
2. Adaptive execution – how contexts affect the ways in which **users** can **find and enact workarounds** that make an intervention and its components a workable proposition in practice.
3. Negotiating capacity- how contexts affect the extent that intervention and its components can **fit** or be **integrated** **into existing ways of working** by their users.
4. Reframing institutional logics – how existing **social structural and social cognitive resources** shape the implementation environment.

Implementation mechanisms

1. Coherence building – how people work together in everyday settings to **understand and plan the activities** that need to be accomplished to put an intervention and its components into practice.
2. Cognitive participation – how people work together to create **networks of participation and communities of practice** around interventions and their components.
3. Collective action – how people work together to **enact interventions** and their components.
4. Reflexive monitoring – how people work together to **appraise interventions** and their components.

Implementation outcomes

1. Intervention performance – what **practices have changes** as a result of interventions and their components being operationalised, enacted, reproduced, over time and across settings.
2. Relational restructuring – how working with interventions and their components have **changed the ways people are organised** and relate to each other.
3. Normative restructuring –how working with interventions and their components have **changed the norms, rules and resources** that **govern action**.
4. Sustainment (normalisation) - how interventions and their components have become **incorporated into practice**.
