# Supplementary material for: Using normalisation process theory to evaluate the implementation of a digital health intervention in community and secondary care long COVID clinics
Source: BMJ Open. 2024 Nov 27;14(11):e092824. doi: 10.1136/bmjopen-2024-092824 (PMC11603829; doi:10.1136/bmjopen-2024-092824)
Supplement: Supplementary file 2 [file bmjopen-2024-092824.reviewer_comments.pdf]

PEER REVIEW HISTORY

BMJ Open publishes all reviews undertaken for accepted manuscripts. Reviewers are asked to complete a checklist review form and are provided with free text boxes to elaborate on their assessment. These free text comments are reproduced below.

ARTICLE DETAILS

Title (Provisional)

Using normalisation process theory to evaluate the implementation of a digital health intervention in community and secondary care long COVID clinics

Authors

Stevenson, Fiona A; Pfeffer, Paul; Walker, Sarah; Ismaila, Hadiza; Jegatheesan, Vinosh; Mohammad, Ibrahim; Blandford, Ann; Linke, Stuart; Hurst, John R.; Ricketts, William; Hamilton, Fiona L; Sunkersing, David; Bradbury, Katherine; Goodfellow, Henry

---

VERSION 1 - REVIEW

**Reviewer**	**1**
**Name**	**Simons, Megan**
**Affiliation**	**Queensland Children's Hospital, Occupational Therapy**
**Date**	**09-Sep-2024**

COI **I have no conflicting or competing interests (which could be personal, financial, intellectual, professional, political or religious in nature) to declare.**

This manuscript explores the implementation of a digital health intervention, The Living With COVID Recovery (LWCR) informed by Normalisation Process Theory. Please consider the following:

- As someone unfamiliar with the DHI, I am unsure from the description provided whether it is an App that the patient voluntarily accesses, or do they access at a set time/frequency etc. Please provide more clarifying details, including how are they oriented to the App. Also, was there a centralised core of AHPs overseeing the roll-out or was it the software developers or research group?

- Methods: please clarify that the 55.5 hours was the total dataset, comprised of 43.5hrs (clinicians and researchers), 2 hours (trust meetings) and 10 hours (core team).

- Please provide more details regarding costs and whether tailoring of the App incurred costs for local services

- Please provide more details within the manuscript of who completed the analysis - details are only currently available in CoreQ questionnaire

- Please provide more detail about how the clinician feedback was shared with other users interested in the DHI (e.g., was it by the software developers?)

- p 16, line 10 - please clarify if the 'intense discussion' was between consumers and clinicians, or the developers and clinicians (for example).

- HCP (p22, line 22) - has this been spelt out in full first? Apologies if I have missed it

**Reviewer**	**2**
**Name**	**HANTEM, Aziz**
**Affiliation**	**Ibn Zohr University**
**Date**	**23-Sep-2024**
**COI**	**No**

the research paper presents an interesting topic on 'Using normalisation process theory to evaluate the

implementation of a digital health intervention in

community and secondary care long COVID clinics'. For the purposes of improvement, the authors should consider the following comments:

- Revise the abstract with a focus on the objectives of the study better developed with well-defined research questions. The abstract should be balanced and better structured.

- In terms of context, it would be advisable to:

add the evolution and history of technological innovations in the UK healthcare sector, particularly digital interventions.

Justify the choice of the theory of the standardisation process and the context of the study.

Better define the research question and the sub-questions.

- Theoretical framework:

add an overview of innovation theory and define the concept of technological innovation and its impact on improving the healthcare system.

Explain why normalisation process theory is most appropriate for evaluating the implementation of a digital health intervention in COVID long community and secondary care clinics.

- Methods and materials:

insert a table mentioning the profile of the participants, Place of work, duration of interview...

- Results and discussion:

the Verbatims of the interviews are limited and require particular development.

The word clouds can be used to better discuss the results.

Try to better structure the results and their discussion.

- Conclusion is rather limited

VERSION 1 - AUTHOR RESPONSE

Reviewer: 1
Dr. Megan Simons, Queensland Children's Hospital, The University of Queensland

Reviewer 1 comments	Response
As someone unfamiliar with the DHI, I am unsure from the description provided whether it is an App that the patient voluntarily accesses, or do they access at a set time/frequency etc. Please provide more clarifying details, including how are they oriented to the App. Also, was there a centralised core of AHPs overseeing the roll-out or was it the software developers or research group?	The following has been added to the manuscript to provide clarification:<br><br>Following a clinical assessment for safety and suitability, patients were signed up to use the DHI ('onboarded') by clinical staff, generally an allied health professional. The DHI took the form of a smart phone application (App) downloaded on their phones. Patients could access the App as and when they need it but could also be directed by clinical staff to review specific sections to support individual symptoms or problems. Given the novelty of the clinical area (long COVID) and differences in how NHS clinics were organised to support patients, the DHI was designed to be flexible to fit with structures and needs of the clinics. Flexibility and iterative use and design were key to the development and implementation of the DHI to maximise utility and use.
Methods: please clarify that the 55.5 hours was the total dataset, comprised of 43.5hrs (clinicians and researchers), 2 hours (trust meetings) and 10 hours (core team).	This has been confirmed in the manuscript as requested. We apologise that this was not clear.
Please provide more details regarding costs and whether tailoring of the App incurred costs for local services	A statement has been added on P6 to confirm as stated below:

	Iterative development and tailoring of the DHI did not incur costs for local services.
Please provide more details within the manuscript of who completed the analysis - details are only currently available in CoreQ questionnaire	This has been clarified as below:<br><br>Initial discussions were held within the implementation work package meetings to consider how best to understand implementation of the DHI. A core group of five (FS, DS, EM, VJ, IM) examined three recordings and transcripts and met on multiple occasions to discuss initial ideas and resolve any differences of interpretation. All recordings were then reviewed by the first author, who conducted coding across the whole data set, taking into account earlier discussions regarding the data. Initial analysis was conducted using the principles of reflexive thematic analysis[13], with themes subsequently mapped against the constructs of Normalisation Process Theory (NPT)[14]. The wider project team reviewed the analysis, and changes were made by the first author.
Please provide more detail about how the clinician feedback was shared with other users interested in the DHI (e.g., was it by the software developers?)	Further details have been added about sharing clinical feedback on p16<br><br>Clinicians were also invited to contribute to a feedback loop for iterative development in the context of a rapidly evolving understanding of long COVID, allowing them to shape the DHI and use it in ways that best suited their clinical environment and the organisation of their services.
p 16, line 10 - please clarify if the 'intense discussion' was between consumers and clinicians, or the developers and clinicians (for example).	The 'intense discussion' was between allied health professionals. This has been clarified as reproduced below:<br><br>Management of this in practice formed a point of intense discussion between AHPs who had adopted the DHI and their colleagues relating to giving patients access to information in the DHI which was not filtered on a patient-by-patient basis by a medical professional
HCP (p22, line 22) - has this been spelt out in full first? Apologies if I have missed it	This was previously spelt out in full on p6

Reviewer: 2
Prof. Aziz HANTEM, Ibn Zohr University

Reviewer 2 comments	Response
Revise the abstract with a focus on the objectives of the study better developed with well-defined research questions. The abstract should be balanced and better structured.	The objectives have been rewritten in the abstract and abstract reviewed in relation to structure. The has been reproduced below.<br><br>**Abstract**<br><br>**Objectives**<br>The potential and expected benefits of digital health interventions have long been discussed, yet substantial challenges are associated with deploying digital health interventions (DHI) at scale. Insights are presented concerning the implementation of a digital health intervention consisting of a patient-facing app and a digital dashboard for clinicians providing supported self-management for long COVID to support both clinicians and patients.<br>**Design**<br>Qualitative reflexive thematic analysis, mapped against Normalisation Process Theory.<br>**Setting**<br>Fifty-five and a half hours of zoom recordings of meetings between clinicians in community and secondary care long COVID clinics and members of the research team.<br>**Participants**<br>Allied health professionals, service delivery managers, and members of the core team, including representatives from industry partners.<br>**Results**<br>The DHI fitted with contextual circumstances and the design supported flexibility to suit circumstances in different trusts. The DHI also aligned with existing ways of working.<br>Healthcare professionals worked together to support the implementation of the DHI, requiring flexibility to take account of local circumstances. The DHI was appraised in both positive and negative terms by healthcare professionals. Using DHIs was said to have the potential to complement care but not be a replacement for face-to-face clinical input. The DHI was judged to

	have demonstrated the potential to affect long-established patterns and organisational structures of engagement between healthcare professionals and patients in terms of access to care.<br>**Conclusions**<br><br>NPT provided a framework for considering both individual agency and the organisation context, enabling reflections to be made at the level of the structure of services as well as people's experiences. The discipline of considering first the context, then the work and finally the practical effects helped place order on the 'mess' involved in the rapid cycle of developing, refining, and implementing a DHI in an atypical environment (a pandemic).
In terms of context, it would be advisable to add the evolution and history of technological innovations in the UK healthcare sector, particularly digital interventions.	A short statement has been added based on the recent Darzi report in the UK which commented on digital interventions in the NHS. Please see below for the additional text:<br><br>The potential and expected benefits of digital health interventions have long been discussed. However, the UK NHS has recently been judged to be 'in the foothills of digital transformation' with the benefits not fully realised and interventions adding to the workload of clinicians rather than releasing more time to care[1]. Moreover, there are substantial challenges with deploying digital health interventions at scale[,2,3,4]. The COVID-19 pandemic resulted in the rapid adoption of digital technology in the NHS and significant changes in the development, reconfiguration, and delivery of services more widely[5]. These rapid changes have shown what is possible, although recent data indicates that post pandemic most appointments are again being delivered face-to-face[6].
In terms of context it would be advisable to Justify the choice of the theory of the standardisation process and the context of the study.	Further details have been added to justify normalisation process and the context of the study in the methods section:

	NPT as an established theory of implementation developed as a tool to explain and evaluate the processes that shape the translation of innovations in the organisation and delivery of healthcare[14]. It offers a coherent and stable set of explanations of implementation processes and characterises the mechanisms that motivate and shape these processes and so can be used to evaluate and understanding implementation processes[15]. Its use was planned from the inception of the study and shaped data collection and analytic direction throughout the project by providing a structure to support capturing of changes in the way people thought about and used the DHI as it was implemented into practice that could then be used to further iterate the DHI for future users.
In terms of context better define the research question and the sub-questions.	This has been revisited and refined at the end of the background section:<br><br>We present insights concerning the implementation of LWCR DHI into clinical care to support both clinicians and patients, while simultaneously offering opportunities for more efficient delivery of services. Specifically, we were interested in how decisions in relation to implementation of a digital health intervention providing supported self-management for long COVID into clinical care to support both clinicians and patients were negotiated and sustained.
Add an overview of innovation theory and define the concept of technological innovation and its impact on improving the healthcare system.	We have not added an overview of innovation theory as this is not the focus of this work. Innovation theory explains how new ideas lead to advancements in society, technology, and business, while our focus, and the reason we chose normalisation process theory (NPT) was to consider how the digital health innovation developed as part of this project resulted in new ways of thinking and working and become part of everyday clinical practice.
Explain why normalisation process theory is most appropriate for evaluating the implementation of a digital health intervention in COVID long community and secondary care clinics.	This is provided on page 10 as follows:<br><br>NPT provided a framework to build on existing knowledge and theory relating to implementation, as well as an opportunity to consider the robustness of the tenets of

	NPT in relation to the extraordinary circumstances and organisational pressures and changes during the COVID pandemic.
insert a table mentioning the profile of the participants, Place of work, duration of interview...	Table 1 outlines the meeting purpose, number of meetings, attendees and the date range during which meetings took place. As stated in the methods all meetings lasted one hour, with one lasting 1.5 hours. As a team we considered providing more details on the profiles of participants but decided against this for fear it would increase the risk of identifying participants. This risk was judged to outweigh any possible benefits of the provision of further details.
The Verbatims of the interviews are limited and require particular development.	The results and verbatim quotations have been reviewed and additional analysis provided throughout the results section (pages 10 - 23)
The word clouds can be used to better discuss the results	We are not sure we have interpreted this comment correctly but on reflection did not see the added value of creating word clouds to elucidate our analysis.
Try to better structure the results and their discussion	The results have been revisited to provide greater clarity of argument.
Conclusion is rather limited	This has been reviewed and further detail added as below:<br><br>Using the tenets of NPT helped us understand and elucidate the organisational changes necessary to implement a DHI to support people with long COVID while understanding of long COVID was evolving, and the health service was experiencing extreme organisational stress.<br><br>We demonstrated how success in implementing a DHI depends on understanding the complexity of the context, fostering cohesive teamwork for execution of the implementation, and evaluating tangible changes resulting from the intervention.<br><br>NPT helped us frame our analysis to understand the requirement for people to work differently at all levels of the organisation to implement the LWCR DHI.

Commented [AB1]: ...and?

	We were able to capture the 'workarounds' people used and the necessity of champions, but also a willingness to engage people across different disciplines to work together to make implementation happen.<br><br>Crucially, even in times of extreme organisational change, issues such as managing information governance and problems with integrating data collected via the DHI into patient records remained. Analysis demonstrated the fragmentation of clinical services and differing possibilities across NHS services, with negotiation necessary at the level of each trust reflecting the difficulties, even in times of crisis, of implementing services at speed and scale. This paper therefore provides an opportunity to reflect on entrenched issues with implementation associated with the fragmentation of the UK health service.

VERSION 2 - REVIEW

**Reviewer**	**2**
**Name**	**HANTEM, Aziz**
**Affiliation**	**Ibn Zohr University**
**Date**	**26-Oct-2024**
**COI**	

the authors have responded to the various comments and opinions, while respecting research ethics.
